# Association of Epithelial Mesenchymal Transition with prostate and breast health disparities

**DOI:** 10.1371/journal.pone.0203855

**Published:** 2018-09-10

**Authors:** Liza J. Burton, Ohuod Hawsawi, Quentin Loyd, Veronica Henderson, Simone Howard, Maxine Harlemon, Camille Ragin, Robin Roberts, Nathan Bowen, Andrew Gacii, Valerie Odero-Marah

**Affiliations:** 1 Center for Cancer Research and Therapeutic Development, Department of Biological Sciences, Clark Atlanta University, Atlanta, GA, United States of America; 2 African Caribbean Cancer Consortium, Philadelphia, PA, United States of America; 3 Fox Chase Cancer Center, Temple Health, Philadelphia, PA, United States of America; 4 University of West Indies School of Clinical Medicine and Research, Nassau, The Bahamas; 5 Department of Lab Medicine, Kenyatta National Hospital, Nairobi, Kenya; University of South Alabama Mitchell Cancer Institute, UNITED STATES

## Abstract

African Americans (AA) have higher death rates due to prostate and breast cancer as compared to Caucasian Americans (CA), and few biomarkers have been associated with this disparity. In our study we investigated whether epithelial-mesenchymal transition (EMT) with a focus on Snail and Cathepsin L (Cat L), could potentially be two markers associated with prostate and breast health disparities. We have previously shown that Snail can increase Cat L protein and activity in prostate and breast cancer. Western blot and real-time PCR analyses showed that mesenchymal protein expression (Snail, vimentin, Cat L) and Cat L activity (shown by zymography) was higher in AA prostate cancer cells as compared to CA normal transformed RWPE-1 prostate epithelial cells, and androgen-dependent cells, and comparable to metastatic CA cell lines. With respect to breast cancer, mesenchymal markers were higher in TNBC compared to non-TNBC cells. The higher mesenchymal marker expression was functionally associated with higher proliferative and migratory rates. Immunohistochemistry showed that both nuclear Snail and Cat L expression was significantly higher in cancer compared to normal for CA and Bahamas prostate patient tissue. Interestingly, AA normal tissue stained higher for nuclear Snail and Cat L that was not significantly different to cancer tissue for both prostate and breast tissue, but was significantly higher than CA normal tissue. AA TNBC tissue also displayed significantly higher nuclear Snail expression compared to CA TNBC, while no significant differences were observed with Luminal A cancer tissue. Therefore, increased EMT in AA compared to CA that may contribute to the more aggressive disease.

## Introduction

Prostate cancer is the most common non-cutaneous cancer in men and is considered the second most frequent cause of death in men in the United States [1]. Moreover, African American men (AA) have a higher incidence rate and two-fold increased mortality due to prostate cancer as compared to Caucasian American men (CA) [2]. Among the Caribbean population, prostate cancer is the leading cause of cancer death in men, followed by lung cancer [3]. Although most studies in health disparity focus on genomics, some have suggested that this health disparity could also be due to biological factors. Triple-negative breast cancers (TNBCs) are defined as tumors lacking the expression of estrogen receptor-alpha (ER-α), progesterone receptor (PR) and human epidermal growth factor receptor-2 (HER-2), which accounts for approximately 15% of total breast cancer patients, and is more prevalent among young African, African-American and Latino women patients [4]. The mortality due to TNBC is higher in African-American and Latino patients, compared to other ethnic groups [5]. TNBCs are considered to be among the most drug resistant, virulent and difficult to treat subtype of breast cancer and often associated with the Epithelial Mesenchymal Transition (EMT) and a high propensity for early metastasis [6]. The primary cause of breast cancer death is metastasis, which is regulated by several factors and signaling pathways such as EMT, a dynamic process that promotes cell motility with decreased adhesive ability between cells to their neighbors [7].

Epithelial Mesenchymal Transition (EMT) plays a key role in cancer progression and metastasis. Epithelial cells undergo morphological and biochemical changes to form a new mesenchymal phenotype, and can be induced by various signaling molecules such as Transforming Growth Factor-β (TGF-β) and Wnt/β-catenin [8] [9]. EMT can be regulated also by Snail transcription factor, a downstream effector of TGF-β, which leads to down-regulation of epithelial markers such as E-cadherin (tight junction protein), and increase in mesenchymal markers as vimentin and fibronectin [10]. Snail, a zinc finger protein, can induce EMT by binding to E-box elements of E-cadherin promoter to decrease its transcription [11]. Increased Snail expression has been shown to present a high risk for cancer progression [12, 13].

Cathepsin proteins are proteolytic enzymes. There are around 11 human cathepsins expressed at different levels within the human genome. The three major subgroups are based on the amino acid present at the active site; cysteine cathepsins (B,C,H,F,K,L,O,V,W), aspastate cathepsins (D and E), and serine cathepsins (G) [14]. It has been proposed the cathepsins proteins activated in the acidic environments [15]. Cathepsin L (Cat L) is responsible for the degradation of the extracellular matrix and basement membranes [16]. Cat L can also regulate the cell cycle through regulating the nuclear transcription factors [17]. Cat L is reported to be up-regulated in various malignant carcinoma; breast [18], colon [19], prostate [20], head and neck, and lung [21].

We have shown previously that Snail overexpression can upregulate Cat L expression and activity *via* the signal transducer and activator of transcription 3 (STAT3) signaling pathway [22]. We also showed that Snail promotes nuclear translocation of Cat L which subsequently degrades its substrate, Cux1, leading to Cux1 repression of E-cadherin and induction of Snail transcription to further promote EMT [23].

There is scarcity of data on EMT in health disparities, and it has not been studied in the Caribbean population. It was previously published that Kaiso, an EMT inducer, is higher in AA prostate and breast cancer patients as compared to CA [24–27]. Moreover, it was recently published that AA prostate cancer patients displayed higher serum levels of TGF-β3 protein compared to CA patients [28]. However, Snail and Cat L have not been extensively studied with regards to health disparities in prostate and breast cancer.

In this study, we investigated the association of EMT with health disparities, focusing on Snail and Cat L as potential biomarkers. We compared the expression of Snail and Cat L expression AA and CA cells lines as well as patient tissue samples from AA, Caribbean and CA men, as well as biological activity looking at cell proliferation and migration. We observed higher EMT in AA compared to CA along with increased proliferation and/or migration.

## Material and methods

### Reagents and antibodies

Anti-mouse α-tubulin antibody was from Sigma-Aldrich, Inc., St Louis, MO. Rat monoclonal anti-Snail and HRP-conjugated goat anti-rat antibodies were from Cell Signaling Technology, Inc., Danvers, MA. Goat monoclonal anti-Cat L and goat monoclonal anti-vimentin antibodies were purchased from R&D Systems (Minneapolis, MN). The HRP conjugated donkey anti-goat, anti-mouse Twist, and anti-mouse E-cadherin were purchased from Santa Cruz Biotechnology (Santa Cruz, CA). HRP-conjugated sheep anti-mouse and sheep anti-goat were purchased from Amersham Biosciences, Buckinghamshire, UK. Luminata Forte The anti-mouse Occludin antibody was purchased from Invitrogen (Carlsbad, CA). HRP chemiluminescence detection reagent was purchased from EMD Millipore (Billerica, MA). The protease inhibitor cocktail was from Roche Molecular Biochemicals, Indianapolis, IN.

### Cell lines and cell culture

Prostate cells obtained from ATCC, Manassas, VA included RWPE-1 (CA; normal transformed prostate epithelial), LNCaP (derived from lymph node metastasis in CA prostate cancer patient; it is tumorigenic but not metastatic in mice), 22Rv1 (CA; derived after castration-induced regression and relapse of the parental androgen-dependent CWR22, it is tumorigenic but not metastatic in mice), DU145 (derived from brain metastasis in CA male; it is tumorigenic and metastatic in mice), C4-2 (CA; tumorigenic and osseous metastatic subline of LNCaP), PC3 (derived from bone metastasis of CA patient with grade IV PCa; tumorigenic and osseous metastasis in mice), MDA-PCa-2b (derived from bone metastatic site in AA; tumorigenic but not metastatic in mice). E006AA (derived from AA with gleason 6 organ-confined PCa; low tumorigenicity and not metastatic in mice) and E006AA-hT (AA, highly tumorigenic subline of E006AA, not metastatic in mice) were provided as a kind gift from Dr. Shahriar Koochekpour, Roswell Park Cancer Institute, New York, NY. Although E006AA-hT was originally reported as an AA prostate cancer cell line, ATCC ran an STR profile for the original starting material, and found that the results match the original characterization data, however the STR profile was also was found to match the STR profile (86% match) of another renal cell carcinoma cell line even though the originating laboratory did not use the renal carcinoma cell line. ARCaP-epithelial (ARCaP-E, CA, metastatic) and ARCaP-mesenchymal (ARCaP-M, CA, highly metastatic) human prostate cancer cells were a kind gift from Dr. Leland Chung, Cedars-Sinai Medical Center, Los Angeles, CA. The human breast cancer cells lines were obtained from ATCC, Manassas, VA. MCF-7 and T47-D are ER+PR+HER2- (luminal A); MDA-MB-361 and BT-474 are ER+PR+HER2+ (luminal B); MDA-MB-231, HS-578T, BT-549, MDA-MB-468 and HCC-1806 are TNBC models. MCF-7 cells stably transfected with empty Neo vector (MCF-7 Neo) or constitutively active Snail (MCF-7 Snail) were generated previously [29]. All cells except MDA-PCa-2b were grown in RPMI or DMEM (Lonza, Alpharetta, GA) supplemented with 10% fetal bovine serum (FBS) (Atlanta biological, Flowery Branch, GA) and 1X penicillin-streptomycin (Mediatech, Herndon, VA). MDA-PCa-2b cells were grown in HPC1 media with 20% FBS. All cells were maintained at 37°C with 5% CO_2_ in a humidified incubator.

### Western blot analysis

Cells were lysed in a modified RIPA buffer as described previously [29]. Supernatants were collected and quantified using a micro BCA assay (Promega, Madison, WI). 30 μg of cell lysate was resolved using 10% sodium dodecyl sulfate–polyacrylamide gel electrophoresis, followed by trans-blotting onto nitrocellulose membrane (Bio-Rad Laboratories, Hercules, CA). Membranes were incubated with appropriate primary and secondary antibody, followed by visualization using Luminata Forte ECL reagent. The membranes were stripped using Restore western blot stripping buffer (Pierce Biotechnology, Rockford, IL) prior to reprobing with a different antibody.

### Real-time PCR (RT-PCR)

Total RNA was isolated by using an RNeasy Mini Kit (Qiagen, Valencia, CA). Gene expression was defined as the threshold cycle number (C_T_). Mean fold change in expression of the target genes were calculated using the comparative C_T_ method (RU; 2^-ΔCt^). All data were normalized to the quantity of RNA input by Glyceraldehyde 3-phosphate dehydrogenase (GAPDH). The following primers were used Snail (Forward 5’-CACTATGCCGCGCTCTTTC-3’; Reverse 5’-GCTGGAAGGTAAACTCTGCTGGATTAGA-3’, Cat L (Forward 5’-AATACAGCAACGGGCAGCA-3’; Reverse 5’-AGCGGTTCCTGAAAAAGCCR-3’), E-cadherin (Forward 5’-GAAGGTGACEAGAGCCTCTGGAT-3’; Reverse 5’-GATCGGTTACCGTGATCAAAATC-3’), Vimentin (Forward 5’-GCAAAGATTCCACTTTGCGT-3’; Reverse 5’-GAAATTGCAGGAGGAGA-3’)

### Zymography

We utilized the cathepsin zymograpy technique as described previously [30]. Briefly, 1 ml of conditioned media containing 0.1 mM leupeptin was concentrated utilizing vivaspin tube (GE Health Care, Piscataway, NJ). The concentrated conditioned media was diluted 10-fold in 1X RIPA buffer followed by determination of the protein concentration, electrophoresis using 0.2% gelatin substrate (Scholar Chemistry, Rochester, NY), incubation in cathepsin-renaturing buffer (65 mM Tris buffer, pH 7.4 with 20% glycerol) and overnight incubation in pH 6 sodium phosphate assay buffer (0.1 M sodium phosphate buffer, 1 mM ethylenediaminetetraacetic acid, 2 mM dithiothreitol) at 37°C. The gel was stained with Coomassie blue stain (10% acetic acid, 25% isopropanol, 4.5% Coomassie Blue), destained (10% isopropanol and 10% acetic acid) and proteolytic activity visualized as cleared bands. The pH conditions used will show both Cat L and cathepsin S (Cat S) activity.

### Cell migration

We utilized Costar 24-well plates containing a polycarbonate filter insert with an 8- μm pore size, to coat with 4.46 μg/ μl rat tail collagen I (BD Bioscience, Bedford, MA) on the outside for 24 h at 4°C. 5×10^4^ cells were plated in the upper chamber containing RPMI supplemented with 0.1% fetal bovine serum (FBS), whereas the lower chamber contained RPMI supplemented with 10% FBS. After 5 h, cells that migrated to the bottom of the insert were fixed, stained with 0.05% crystal violet, and counted to obtain the relative migration. Each experiment was done in triplicate, and the graphs represent an average of the 3 wells. All the experiments were repeated at least three times.

### Cell viability assay

LNCaP, C4-2, ARCaP-E, ARCaP-M, E006AA, E006AA-hT, MDA-PCa-2b cells were plated at a density of 2,000 cells per well in 96-well plates and allowed to attach overnight. Viability was assessed daily using the CellTiter 96® Aqueous One Solution Cell Proliferation Assay according to the supplier's protocol.

### Ethics statement

This study was approved by the Clark Atlanta University Institutional Review Board, and approved it as of exempt since we used archival samples in which the patients could not be identified.

### Prostate patient samples

AA (36 normal, 38 cancer cases) and CA (33 normal, 45 cancer cases) prostate cancer tissues (formalin-fixed) were purchased from Fox Chase Cancer Center Biosample Repository Facility that provides well-annotated, fully-consented diseased and normal samples collected under an IRB-approved protocol to industry and academic investigators for research purposes. For AA men, the age range was 44–70, with an average age of 57, while for CA, the age range was 47–79, with an average age of 59. We also acquired formalin-fixed patient tissue from the Bahamas (35 normal, 25 cancer cases that was similarly collected under an IRB-approved protocol. For Bahamas tissue, the age range was 47–88, with an average age of 69. The cancer cases ranged from Stage 1 to 4.

### Breast patient samples

Breast patient tissues (formalin-fixed) were purchased from Fox Chase Cancer Center Biosample Repository Facility. The following is the breakdown of the tissue obtained: For AA: normal (n = 24), luminal A (n = 7), TNBC (n = 15). For CA: normal (n = 10), luminal A (n = 14), TNBC (n = 13). The luminal A subtype represents ER+PR+HER2+; TNBC subtype represents ER-PR-HER2-. The ages of CA patient tissue obtained ranged from 36–82, with an average age of 55. The ages of AA patient tissue obtained ranged from 26–82, with an average age of 54.

### Immunhistochemistry

Examination of the expression and distribution of Snail and Cat L was performed by immunohistochemistry (IHC) using prostate tissue (AA and CA normal and cancer varying from Stage 1 to 4) purchased from the Fox Chase Cancer Center, as well as Bahamian patient tissue. IHC was performed using the Avidin-biotin immunohistochemical method. The slides were deparaffinised in xylene and rehydrated using alcohol. Endogenous peroxidase activity was blocked by 3% hydrogen peroxide. After antigen retrieval, sections were incubated with 10% serum to avoid the non-specific binding. Sections were incubated with 1:200 primary antibody against Cat L or Snail at 4°C overnight followed by biotinylated secondary antibody, and incubation with avidin-biotin complex (Vector, Burlingame, CA). Immunoreactivity was visualized using diaminobenzidine (Sigma-Aldrich, St. Louis, MO, USA). The slide was subsequently counterstained with hematoxylin and mounted with xylene solution. Images were acquired using the Axiovision Rel 4.8. Tissue was analyzed and digitally scored using the Leica biosystem software (Digital Histology Shared Resource, Vanderbilt University, Nashville TN), confirmed using our own Aperio Versa Image Scanner and scoring verified by a pathologist.

### Immunofluorescence

5×10^3^ cells were plated into 16 well chamber slides (Bio-Tek, Nunc, Winooski, VT). For treatments, cells were either untreated, treated with 5 or 20 μm Z-FY-CHO for 3 days. Fixation was performed with methanol/ethanol 1:1 volume for 5 min, followed by washes with 1× PBS and blocking with protein blocking solution without serum (Dako, Camarillo, CA) for 10 min at room temp. Subsequently, slides were incubated with primary antibody at 1 1:100 dilution in Dako antibody diluent solution for 1 hr. at room temp. Slides were washed with 1× TBS-T (Dako, Camarillo, CA), then incubated with secondary antibody in the dark for 1 hr. at room temp. Secondary antibodies used were anti-rabbit Oregon green 488 or anti-mouse Alexa red 594 (Invitrogen, Carlsbad, CA). Slides were washed with 1× TBS-T and double deionized water, prior to counterstaining with DAPI (1 μg/ml, Santa Cruz Biotechnology, Santa Cruz, CA). Slides were mounted using Fluorogel mounting medium (Electron Microscopy Sciences, Hatfield, PA). Fluorescence microscopy was performed using Zeiss microscope and Axiovision Rel 4.8 software.

### Statistical analysis

Data were analyzed by a paired student's t-test or ANOVA using GraphPad Prism software. For all experiments * means 0.05 > *p* value > 0.01, ** means 0.01 > *p* value > 0.001, and *** means *p* value < 0.001. For IHC, we utilized Welch Two Sample t-test with Wilcoxon rank sum test with continuity correction and 95 percent confidence interval.

## Results

### EMT marker expression in CA and AA cell lines

**Fig 1:**
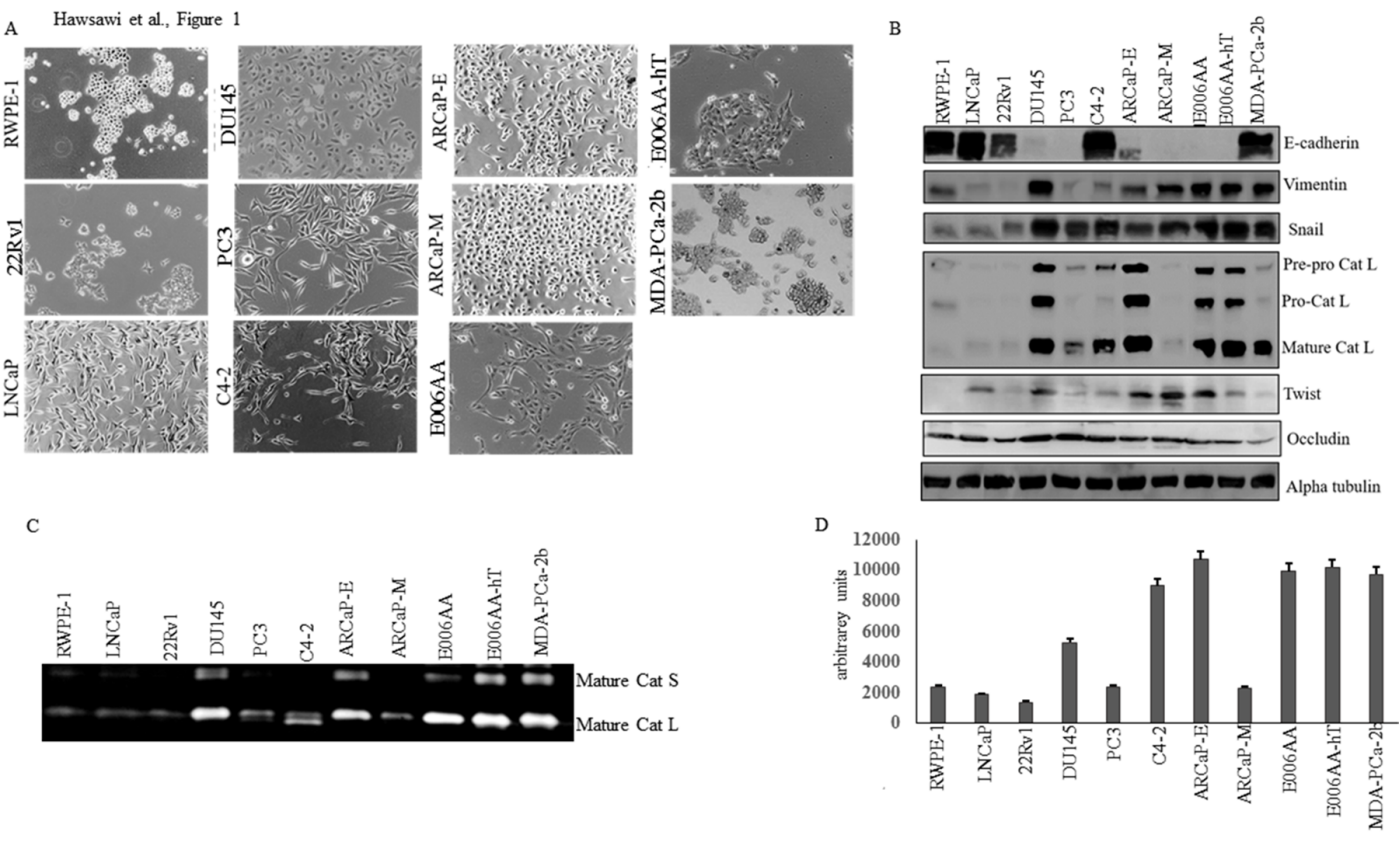
Expression of EMT markers in prostate cell lines. (A) Morphology of Prostate Cancer cell lines. (B)The expression of EMT markers (E-cadherin, vimentin, Snail, Cat L, Twist, and Occludin) were examined by western blot analysis. Alpha tubulin was used as the loading control. (C) Zymogram of Cat L activity in various prostate cell lines. (D) Cat L activity was quantified by densitometry for each band on the gel using NIH image J. (n = 3, *** *p* < 0.001, **p < 0.01, * *p* < 0.05). Values were normalized to untreated controls and the mean± SEM of data were obtained from three independent replicate experiments. Statistical analysis was done with Student *t* test.

**Fig 2:**
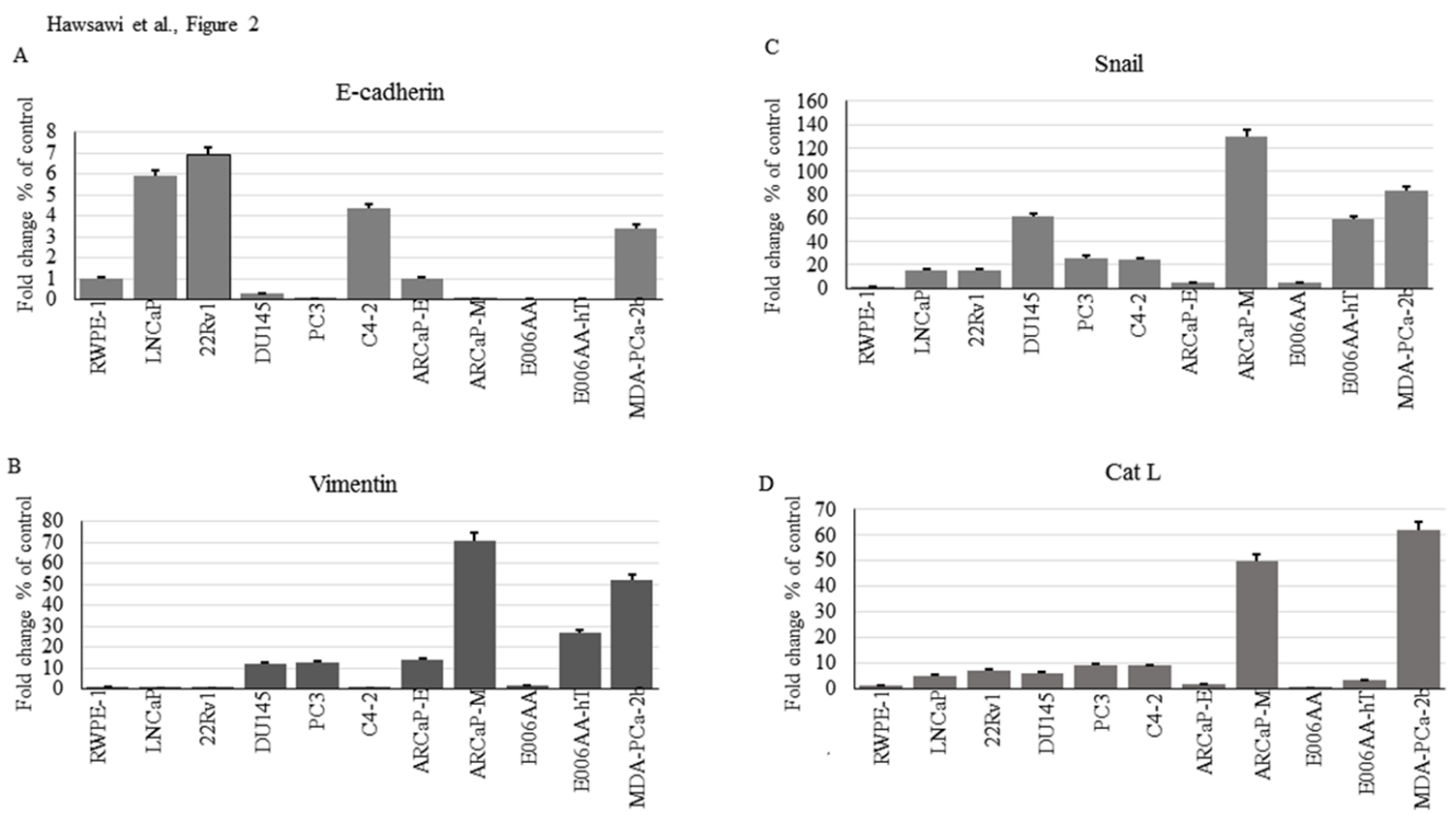
mRNA analysis of EMT markers in CA and AA cell lines. Prostate cell lines were probed for mRNA levels by real-time PCR analysis using primers specific for E-cadherin, Snail, vimentin and Cat L. Results are representative of at least two independent experiments performed in triplicate.

We examined the expression of EMT markers in a panel of prostate cancer cells lines of increasing aggressiveness, and of CA and AA origin, by western blot analysis, as well as Cat L activity by zymography. Epithelial markers examined included E-cadherin and Occludin, while mesenchymal markers included Snail, Cat L, vimentin, and Twist. The morphology of the cell lines is shown in Fig 1A. The normal transformed prostate epithelial cell line (RWPE-1) and the CA tumorigenic but non-metastatic cell lines (LNCaP and 22rV1) expressed low levels of Snail, vimentin, Cat L mesenchymal markers and high levels of E-cadherin (Fig 1B). The metastatic CA prostate cancer cell lines (DU145, PC3, ARCaP-E, ARCaP-M) as well as the tumorigenic but non-metastatic AA cell lines (E006AA, E006AA-hT, MDA-PCa -2b) expressed higher levels of Snail, vimentin, Cat L, Twist and lower levels of E-cadherin (with the exception of C4-2 and MDA-PCa-2b which still expressed high E-cadherin) (Fig 1B). Occludin levels did not appear significantly different between the cell lines (Fig 1B). RWPE-1, 22RV1, LNCaP, PC-3 and ARCaP-M had a low amount of mature Cat L activity compared to ARCaP-E, C4-2, DU145, and the AA cell lines, which displayed the highest amount of mature Cat L activity (Fig 1C and 1D). Real time PCR analysis largely supported the western blot data with some differences including ARCaP-E and E006AA displaying low levels of Snail and Cat L mRNA (Fig 2) despite the protein being more abundant (Fig 1B) which may suggest some post-translational regulations. Therefore, the data suggests that there is more EMT in CA metastatic cell lines as compared to non-metastatic cell lines, and that non-metastatic AA cell lines also display EMT at levels similar to aggressive CA cell lines.

### AA cell lines have increased migration and cell viability comparable to metastatic CA cell lines

**Fig 3:**
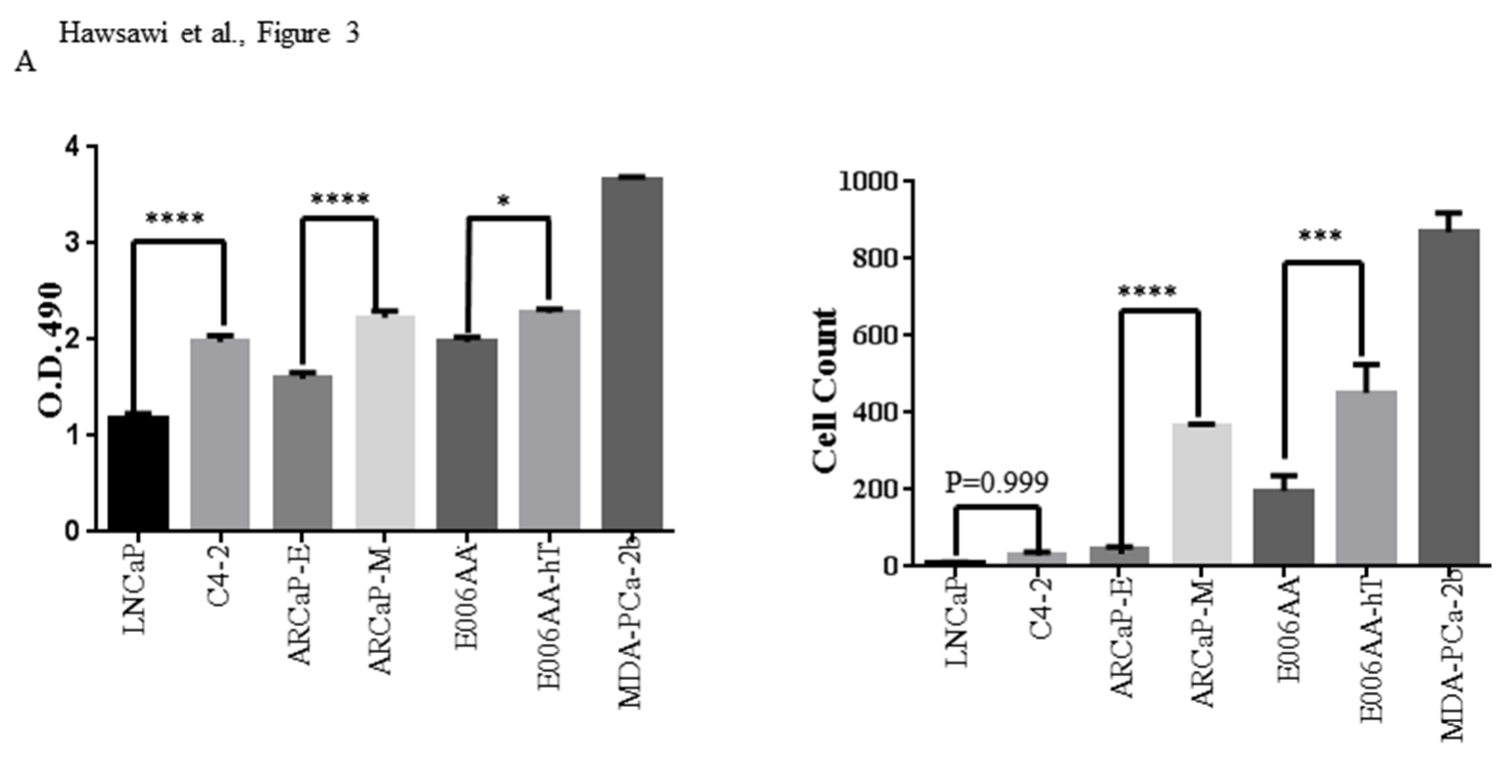
AA cell lines are highly proliferative and migratory. CA (LNCaP, C4-2, ARCaP-E, ARCaP-M) and AA (E006AA, E006AA-hT, MDA-PCa-2b) cells were analysed for (A) cell viability using MTS and (B) transwell migration across a collagen-coated boyden chamber. (*** *p* < 0.001, * *p* < 0.05). Mean± SEM of data were obtained from three independent replicate experiments. Statistical analysis was done with Student *t* test.

To further examine EMT biological functions *in vitro*, we looked at cell viability and migration in the different cell lines. As compared to LNCaP, AA non-metastatic cell lines displayed higher cell viability and migration, comparable or even higher than some of the metastatic CA cell lines (Fig 3A and 3B). MDA-PCa-2b showed the highest proliferative and migratory rates (Fig 3A and 3B).

### TNBC cell lines display more EMT and cell migration compared to non-TNBC cells

**Fig 4:**
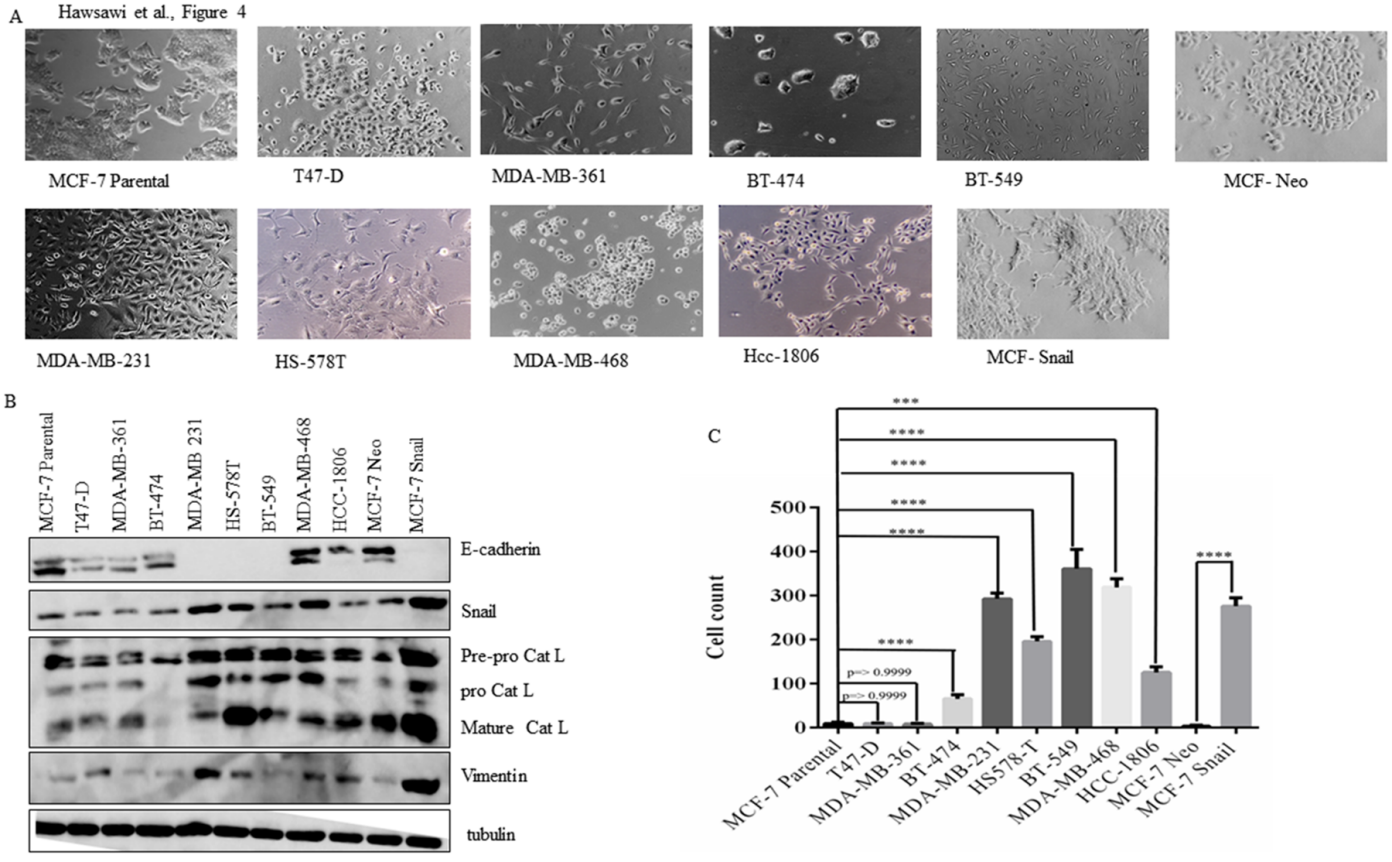
TNBC cell lines are more mesenchymal, proliferative and migratory as compared to non-TNBC cell lines. (A) The morphology of estrogen-receptor (ER)-positive or non-TNBC cell lines (MCF-7, T47-D, MDA-MB-361 and BT-474) and TNBC cell lines (MDA-MB-231, HS-578T, BT-549, MDA-MB-468 and HCC-1806) are shown. (B) TNBC and non-TNBC cells were analyzed for EMT marker expression by western blot analysis. Alpha tubulin was used as the loading control. (C) transwell migration across a collagen-coated boyden chamber was performed. (*** *p* < 0.001, **** *p* < 0.0001). Mean± SEM of data were obtained from three independent replicate experiments. Statistical analysis was done with Student *t* test.

Since mortality due to TNBC is higher in African-American and Latino patients, compared to other ethnic groups [5], and is associated with EMT, we wanted to confirm this using a panel of breast cancer cell lines whose morphology is shown in Fig 4A. Western blot analysis showed higher Snail and vimentin expression in TNBC cell lines (MDA-MB-231, HS-578T, BT-549, MDA-MB-468 and HCC-1806) compared to Luminal cell lines (MCF-7, T47-D, MDA-MB-361 and BT-474) (Fig 4B) which was associated with higher cell migration (Fig 4C), suggestive of EMT. Mature Cat L was also higher in TNBC cells as compared to non-TNBC cells (Fig 4B). E-cadherin was also generally lower in TNBC cells except for MDA-MB-468 and HCC-1806 cells which still expressed E-cadherin in addition to high Snail and vimentin (Fig 4B). Additionally, overexpression of Snail in MCF-7 cells promoted EMT and increased Cat L expression (Fig 4B and 4C).

### Snail and Cat L expression in prostate patient tissue of AA, Bahamas and CA origin

**Fig 5:**
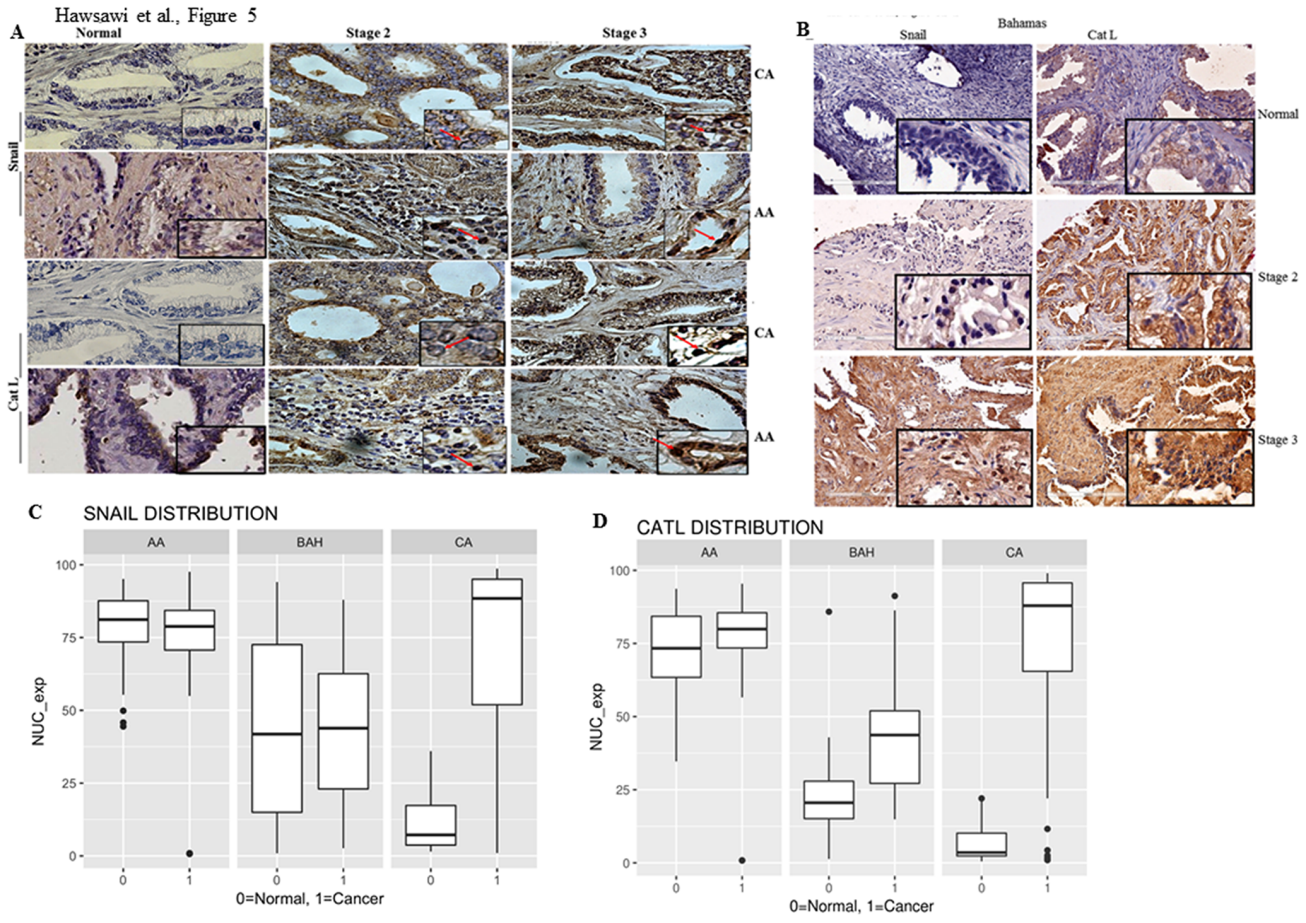
Snail and Cat L expression in prostate patient tissue. (A) Immunohistochemical (IHC) analysis was performed using normal and tumor matched prostate adenocarcinoma tissue from Caucasian American (CA) African American (AA) men. For AA: n = 36 normal, n = 38 cancer and for CA: n = 33 normal, n = 45 cancer. Representative images are shown at 20X. (B) IHC for Snail and Cat L was similarly performed for Bahamian (BAH) men normal and tumor-matched tissue (n = 34 normal, n = 22 cancer) and representative images shown at 20X. Inset, 40X mag. Nuclear staining for (C) Snail and (D) Cat L was digitally scored using Image Scanner and verified by a Pathologist and graphed. Points represent nuclear Snail staining intensity of individual CA and AA patients; bars represent the median value for the sample set.

We sought to perform *in vivo* studies using patient tissue, and Snail and Cat L as representative EMT markers. We previously reported that the expression of Cat L in prostate cancer tissue from US Biomax increased with tumor grade and changed its localization from the cytoplasm to the nucleus with increased tumor grade, but in normal tissue levels were low [31]. We also have shown that an increase in Snail expression is correlated with an increase in Cat L activity and expression [22, 23]. We utilized normal/tumor matched AA, CA of varying grades, and included prostate patient tissue from the Bahamas (BAH) to stain for Snail and Cat L. We observed that Snail and Cat L localization was nuclear/cytoplasmic in early stages of prostate cancer (Stage 2) in AA samples compared to CA where these proteins were predominantly cytoplasmic and nuclear localization was observed in Stage 3 (Fig 5A). Representative tissue from the Bahamas showed that Snail and Cat L increased between normal and cancer (Fig 5B). We digitally scored nuclear staining for Snail and Cat L in these tissues and verified staining scores with a Pathologist. Data for nuclear staining was plotted and since we did not have enough tissue in different Stages for statistical significance, we plotted normal vs cancer (all Stages from 1 to 4). This revealed that nuclear Snail and Cat L which have been implicated in EMT were low in CA normal tissue but significantly increased in CA prostate cancer tissue (p = 3.615e-07 for Snail; p = 2.674e-07 for Cat L) (Fig 5C, S1 Table). Interestingly Snail and Cat L were already high in AA normal tissue, higher than normal CA and not significantly different from AA cancer tissue (p = 0.3236 for AA cancer vs normal for Snail and p = 0.08747 for AA cancer vs normal for Cat L) (Fig 5C, S1 Table). When compared to normal CA, normal AA prostate tissue stained significantly higher for both nuclear Snail and Cat L (p = 2.2e-16; S2 and S3 Tables). This may contribute to the aggressive nature of prostate cancer in AA, in that the proteins may already be expressed in normal tissue and at early stages the localization of Cat L and Snail may be key to changes in EMT. The Bahamas tissue staining was intermediate between CA and AA in both normal and cancer tissue, however, there was not statistical difference in Snail expression between Bahamian normal and cancer (p = 0.05622), but Cat L was significantly higher in cancer vs normal (p = 0.0002003) (Fig 5C, S1 Table). Therefore, nuclear Snail and Cat L is increased in cancer regardless of race, and interesting significant differences were observed in normal tissue where nuclear Snail and Cat L was higher in normal AA and Bahamas tissue compared to normal CA tissue.

### Snail and Cat L expression is higher in AA and TNBC cells compared to CA and non-TNBC cells

**Fig 6:**
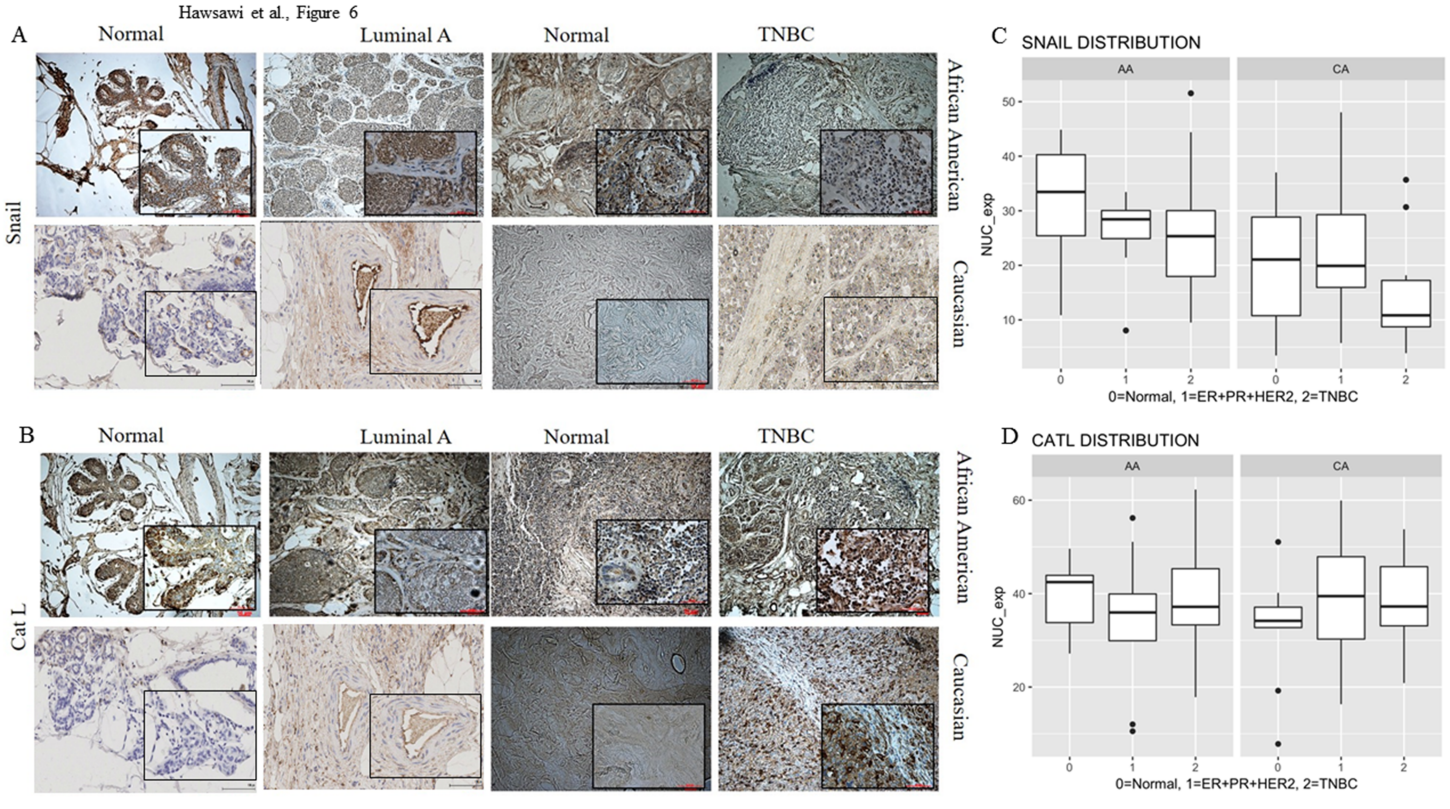
Snail and Cat L expression in breast patient tissue. Immunohistochemical (IHC) analysis was performed using normal, ER-positive or luminal A and TNBC breast tissue from Caucasian American (CA), and African American (AA) women with antibody against (A) Snail or (B) Cat L. For AA: normal (n = 24), luminal A (n = 7), TNBC (n = 15). For CA: normal (n = 10), luminal A (n = 14), TNBC (n = 13). Representative images are shown at 10X. Inset, 20X mag. Nuclear staining for (C) Snail and (D) Cat L was digitally scored using Image Scanner and verified by a Pathologist and graphed. Bars represent the median value for the sample set.

We investigated the expression of Snail and Cat L in breast patient tissue by IHC. Data indicated that normal AA breast epithelial tissue adjacent to luminal A cancer expressed high levels of cytoplasmic Snail and Cat L compared to normal CA (which was mostly low to negative) and comparable to adjacent cancer, while normal AA breast epithelial tissue adjacent to TNBC expressed high levels of nuclear Snail and Cat L similar to TNBC tissue (Fig 6A and 6B). When nuclear staining was scored and plotted, it revealed that AA normal and TNBC tissue displayed significantly higher nuclear Snail (p = 0.0161 for normal and p = 01921 for TNBC) as compared to CA, while no significant differences were observed between races for Luminal A patient tissue (Fig 6C, S4 Table). Although Cat L staining seemed higher in normal AA vs normal CA breast tissue (Fig 6B and 6D), it was not statistically significant (p = 0.1141, S5 Table). Therefore, the most significant racial difference in staining was observed in Snail expression being higher in AA normal and TNBC breast tissue compared to CA tissue.

## Discussion

African American men (AA) have the higher incidence and mortality rate due to prostate cancer compared to Caucasian American men (CA) [2]. Several epidemiological studies have suggested that different factors may contribute to increased prostate cancer risk for AA, but the direct risk factors remain to be elucidated. Few biomarkers have been associated with prostate cancer health disparity. Notably, the epidermal growth factor receptor signaling was found to be more commonly expressed in tumors from AA when compared with European-American patients (EA) [32]. Additionally, several known metastasis-promoting genes, including *AMFR*, *CXCR4* and *MMP9*, were more highly expressed in tumors from AA than EA [33, 34]. In research by Rose *et al*., their data pointed to significant differences in tumor immunobiology and inflammation pathways between AA and EA [35]. Triple-negative breast cancers (TNBCs) that lack expression of estrogen receptor-alpha (ER-α), progesterone receptor (PR) and human epidermal growth factor receptor-2 (HER-2), is more prevalent among women of African descent and Latino women patients [4]. The mortality due to TNBC is higher in African-American and Latino patients, compared to other ethnic groups [5]. This study sought to find novel biological markers associated with health disparities that fall within a similar signaling pathway.

Epithelial Mesenchymal Transition (EMT) is the early step where carcinoma cells become more invasive and metastatic [12]. Snail, transcription factor is well known to play an important role in promoting EMT [36]. In our current study, we show that generally AA cell lines (E006AA, E006AA-hT, MDA-PCa-2b) display EMT markers Snail, vimentin, Twist, Cat L to levels similar to metastatic CA cell lines (DU145, PC3, C4-2, ARCaP), as well as lower E-cadherin as compared to normal (though immortalized) prostate epithelial RWPE-1, and non-metastatic LNCaP and 22Rv1 cell lines. The exception was C4-2 and MDA-PCa-2b that still expressed large amounts of E-cadherin as well as mesenchymal markers suggesting that this cell line represents an intermediate EMT cell; however, these cell lines were still highly proliferative and migratory, as was E006AA and E006AA-hT, as compared to LNCaP cells. Generally, the AA cell lines displayed high cell viability and migration comparable to metastatic CA cell lines, and higher than non-metastatic CA cell lines. For simplicity of comparison, we were comparing AA to CA cell lines based on their tumorigenic/metastatic potential in mice, and not their site of origin. We could have also compared based on site of origin and still we would have concluded that even organ-confined E006AA cells displayed EMT similar to CA cell lines derived from metastatic sites, and higher than RWPE-1 or 22Rv1, although technically, 22Rv1 are a castrate-resistant derivative of parental androgen-dependent CWR22. Additionally, although ARCaP-M has a low protein expression and secreted activity of Cat L compared with ARCaP-E, our previous research has shown that nuclear localization of Cat L is higher in ARCaP-M compared to ARCaP-E and this contributes to more EMT seen in ARCaP-M [23]. Therefore, when looking at Cat L, nuclear expression may be more indicative of EMT than secreted protein activity. It is interesting to note that although E006AA is derived from organ-confined prostate cancer, it still displays EMT similar to metastatic cell lines. The caveat to these studies is the lack of sufficient AA cell lines for studies in prostate cancer, as well as lack of a normal epithelial AA prostate cell line for comparison. Another caveat is the recent characterization by ATCC of E006AA-hT cell line which shows they not only have AA characteristics, but also renal cell carcinoma characteristics, so the results we have obtained with this cell line may pertain to renal cell carcinoma, which are also known to undergo EMT [37]. However, since we also had E006AA and MDA-PCa-2b that are confirmed AA cell lines, from these studies we were still able to glean some useful comparative information which may guide future researchers trying to study EMT, and which cell model may be most suitable. There is one previously published study that compared some EMT markers in various prostate cells, namely LNCaP, C4-2, E006AA and MDA-PCa-2b which similarly suggested that E006AA biomarkers expression is similar to metastatic CA cell lines [38]. This supports our finding that utilized a larger panel of prostate cell lines. A discrepancy with this previous study and ours, is that MDA-PCa-2b was characterized as a low aggressive cell line due to the fact that E-cadherin was high and vimentin was low which was explained in part by the possibility that reversal of EMT known as mesenchymal epithelial transition or MET can occur at bone metastatic site [38], however, although we similarly saw high levels of E-cadherin, we also observed high vimentin expression. The discrepancy may be in part due to differences in culturing or passage number between the two studies. Additionally, our study includes functional cell viability and migration studies that were not performed in the previous study [38]. This shows that although MDA-PCa-2b expresses high E-cadherin, it displays very high proliferative and migratory potential suggesting that it is an aggressive AA cell line.

With regards to breast cancer cells, we took a slightly different approach and compared TNBC to non-TNBC since TNBC is higher in AA and is associated with worse prognostic outcome. Our studies showed that TNBC cell lines (MDA-MB-231, HS-578T, BT-549, MDA-MB-468 and HCC-1806) displayed more EMT and Cat L expression compared to luminal cell lines (MCF-7, T47-D, MDA-MB-361 and BT-474), which was associated with higher cell migration. However, similar to what was observed with MDA-PCa-2b, E-cadherin was still expressed in MDA-MB-468 and HCC-1806 cells suggesting intermediate EMT despite higher cell migration compared to non-TNBC cells. Additionally, overexpression of Snail in MCF-7 cells promoted EMT and increased Cat L expression.

In order to look at some of the EMT markers *in vivo*, we focused on Snail and Cat L. We previously demonstrated that overexpression of Snail in prostate cancer leads to increase the Cat L expression and activity [22]. To induce EMT, Snail transcription factor must localize to the nucleus [39, 40]. Research has also shown that Cat L is localized to the nucleus in different cancers [31, 41–44], which has been associated with decrease survival [19]. We have also previously shown that Snail can promote Cat L translocation into the nucleus where it promotes EMT via proteolysis of its nuclear substrate Cux1, leading to further Snail transcription and decreased E-cadherin transcription [23]. In patient tissue, it was interesting to note that although Snail and Cat L expression was high in prostate cancer tissue from AA, Bahamian, and CA origin, when all the different stages were grouped together, normal tissue from AA also expressed high levels of Snail and Cat L while it was intermediate in Bahamian prostate cancer tissue when compared to normal CA prostate tissue. This may contribute to the aggressive nature of prostate cancer in AA, in that the proteins may already be expressed in normal tissue and at early stages, the localization of Cat L and Snail may contribute to EMT early. Indeed, IHC did show that in some Stage 2 prostate cancer patient tissue, Snail and Cat L was already nuclear in AA, while cytoplasmic in CA, but by Stage 3 both proteins were nuclear. Unfortunately, we did not have large enough patient numbers to graph nuclear expression by grade and get statistical significance, but it would be interesting to also quantify nuclear expression of Snail and Cat L by grade. A previous study looked at Snail, N-cadherin and MMP2 in 15 AA and 15 European American men (EA) and found that these EMT markers were higher in AA compared to EA [38], this differs slightly from our data which showed that nuclear Snail expression was comparably high in CA and AA; this may be explained in part by the fact that we focused only on nuclear Snail while the previous study looked at overall Snail levels that includes cytoplasmic Snail. Our study also included data on normal prostate from CA and AA that was not part of the previous study. With the breast patient tissue, it was similarly interesting to note that overall, AA normal displayed higher levels of nuclear Snail and Cat L compared to normal CA breast epithelial tissue. However, we were not able to recapitulate data that showed that TNBC had higher levels of nuclear Snail and Cat L compared to non-TNBC, probably due to the small sample sizes. We may need larger sample sizes to get significant data for the breast study, though we do observe a trend towards higher nuclear Snail and Cat L in AA compared to CA tissue overall. Although nuclear Cat L has been associated with TNBC [37], this is the first report comparing both nuclear Snail and Cat L in different races.

In conclusion, our study showed that high expression of EMT markers in AA prostate cancer cells lines and TNBC cell lines, as well as high nuclear Snail and Cat L expression in normal AA prostate and breast tissue; this may possibly mediate the EMT transition earlier in AA compared to CA. This correlated with high cell viability and/or migration in AA prostate cancer and TNBC cell lines, comparable to more aggressive CA prostate cancer and non-TNBC cell lines, respectively. As prostate and breast cancer disproportionately affects AA, the discovery of true risk alleles and genes could have important implications for early detection of cancer in this high-risk population.

## Supporting information

S1 Table Comparison of Nuclear Snail and Cat L in prostate normal vs cancer. (DOCX)

S2 Table Comparison of nuclear Snail distribution in AA vs CA prostate patients. (DOCX)

S3 Table Comparison of nuclear Cat L distribution in AA vs CA prostate patients. (DOCX)

S4 Table Comparison of nuclear Snail distribution in AA vs CA breast patients. (DOCX)

S5 Table Comparison of nuclear Cat L distribution in AA vs CA breast patients. (DOCX)

## References

[1] Etzioni R, Penson DF, Legler JM, Di Tommaso D, Boer R, Gann PH, et al Overdiagnosis due to prostate-specific antigen screening: lessons from US prostate cancer incidence trends. Journal of the National Cancer Institute. 2002;94(13):981–90. 12096083 10.1093/jnci/94.13.981

[2] DeSantis CE, Siegel RL, Sauer AG, Miller KD, Fedewa SA, Alcaraz KI, et al Cancer statistics for African Americans, 2016: Progress and opportunities in reducing racial disparities. CA: a cancer journal for clinicians. 2016. 10.3322/caac.21340 26910411

[3] Razzaghi H, Quesnel-Crooks S, Sherman R, Joseph R, Kohler B, Andall-Brereton G, et al Leading Causes of Cancer Mortality—Caribbean Region, 2003–2013. MMWR Morbidity and mortality weekly report. 2016;65(49):1395–400. doi: 10.15585/mmwr.mm6549a3 . 27977639 10.15585/mmwr.mm6549a3

[4] Chen J, Russo J. ER alpha-negative and triple negative breast cancer: molecular features and potental therapeutic approaches. Biochim Biophys Acta. 2009;1796(2):162–75. 10.1016/j.bbcan.2009.06.003 19527773 PMC2937358

[5] Wu Y, Sarkissan M, Elshmali Y, Vadgama J. Triple Negative Breast tumors in African -American and Hispanic/Latina Women are high in CD44+, Low in CD24+ and Have loss of PTEN. Plos one. 2013;8(10):e78259 10.1371/journal.pone.0078259 24167614 PMC3805609

[6] Carey L, Winer E, Viale G, Cameron D, Gianni L. Triple-negative breast cancer: disease entity or title of convenience? Nat Rev Clin Oncol. 2010;7(12):683–92. Epub 2010/09/30. 10.1038/nrclinonc.2010.154 . 20877296

[7] Wang Y, Zhou B. Epithelial-mesenchymal Transition—A hallmark of Breast Cancer metastasis. Cancer Hallm. 2013;1(1):38–49. 10.1166/ch.2013.1004 24611128 PMC3944831

[8] Willis BC, Borok Z. TGF-β-induced EMT: mechanisms and implications for fibrotic lung disease. American Journal of Physiology-Lung Cellular and Molecular Physiology. 2007;293(3):L525–L34. 10.1152/ajplung.00163.2007 17631612

[9] Kim K, Lu Z, Hay ED. DIRECT EVIDENCE FOR A ROLE OF β‐CATENIN/LEF‐1 SIGNALING PATHWAY IN INDUCTION OF EMT. Cell biology international. 2002;26(5):463–76. 12095232 10.1006/cbir.2002.0901

[10] Garg M. Epithelial-mesenchymal transition-activating transcription factors-multifunctional regulators in cancer. World J Stem Cells. 2013;5(4):188–95. 10.4252/wjsc.v5.i4.188 24179606 PMC3812522

[11] Fendrich V, Waldmann J, Feldmann G, Schlosser K, König A, Ramaswamy A, et al Unique expression pattern of the EMT markers Snail, Twist and E-cadherin in benign and malignant parathyroid neoplasia. European Journal of Endocrinology. 2009;160(4):695–703. 10.1530/EJE-08-0662 19176646

[12] Cano A, Pérez-Moreno MA, Rodrigo I, Locascio A, Blanco MJ, del Barrio MG, et al The transcription factor snail controls epithelial–mesenchymal transitions by repressing E-cadherin expression. Nature cell biology. 2000;2(2):76–83. 10.1038/35000025 10655586

[13] Xu J, Lamouille S, Derynck R. TGF-β-induced epithelial to mesenchymal transition. Cell research. 2009;19(2):156–72. 10.1038/cr.2009.5 19153598 PMC4720263

[14] Turk V, Stoka V, Vasiljeva O, Renko M, Sun T, Turk B, et al Cysteine cathepsins: from structure, function and regulation to new frontiers. Biochimica et Biophysica Acta (BBA)-Proteins and Proteomics. 2012;1824(1):68–88. 22024571 10.1016/j.bbapap.2011.10.002 PMC7105208

[15] Turk V, Turk B, Turk D. Lysosomal cysteine proteases: facts and opportunities. The EMBO journal. 2001;20(17):4629–33. 10.1093/emboj/20.17.4629 11532926 PMC125585

[16] Herszényi L, Farinati F, Cardin R, István G, Molnár LD, Hritz I, et al Tumor marker utility and prognostic relevance of cathepsin B, cathepsin L, urokinase-type plasminogen activator, plasminogen activator inhibitor type-1, CEA and CA 19–9 in colorectal cancer. BMC cancer. 2008;8(1):194. 18616803 10.1186/1471-2407-8-194 PMC2474636

[17] Brix K, Dunkhorst A, Mayer K, Jordans S. Cysteine cathepsins: cellular roadmap to different functions. Biochimie. 2008;90(2):194–207. 10.1016/j.biochi.2007.07.024 17825974

[18] Sudhan D, Rabaglino B, Wood C, Siemann D. Role of Cathepsin L in breast cancer angiogenesis. AACR; 2015.

[19] Sullivan S, Tosetto M, Kevans D, Coss A, Wang L, O'donoghue D, et al Localization of nuclear cathepsin L and its association with disease progression and poor outcome in colorectal cancer. International Journal of Cancer. 2009;125(1):54–61. 10.1002/ijc.24275 19291794

[20] Sudhan DR, Pampo C, Rice L, Siemann DW. Cathepsin L inactivation leads to multimodal inhibition of prostate cancer cell dissemination in a preclinical bone metastasis model. International Journal of Cancer. 2016. 10.1002/ijc.29992 PMC4805507 26757413

[21] Chauhan SS, Goldstein LJ, Gottesman MM. Expression of cathepsin L in human tumors. Cancer research. 1991;51(5):1478–81. 1997186

[22] Burton LJ, Smith BA, Smith BN, Loyd Q, Nagappan P, McKeithen D, et al Muscadine grape skin extract can antagonize Snail-cathepsin L-mediated invasion, migration and osteoclastogenesis in prostate and breast cancer cells. Carcinogenesis. 2015;36(9):1019–27. 10.1093/carcin/bgv084 26069256 PMC4643647

[23] Burton LJ, Dougan J, Jones J, Smith BN, Randle D, Henderson V, et al Targeting the Nuclear Cathepsin L-CCAAT-displacement protein/cut homeobox Transcription Factor-Epithelial Mesenchymal Transition Pathway in Prostate and Breast Cancer Cells with Z-FY-CHO Inhibitor. Molecular and Cellular Biology. 2016:MCB. 00297–16. 10.1128/MCB.00297-16 PMC5311241 27956696

[24] Jones J, Wang H, Zhou J, Hardy S, Turner T, Austin D, et al Nuclear Kaiso indicates aggressive prostate cancers and promotes migration and invasiveness of prostate cancer cells. The American journal of pathology. 2012;181(5):1836–46. 10.1016/j.ajpath.2012.08.008 ; PubMed Central PMCID: PMC3483816. 22974583 PMC3483816

[25] Bassey-Archibong BI, Hercules SM, Rayner LGA, Skeete DHA, Smith Connell SP, Brain I, et al Kaiso is highly expressed in TNBC tissues of women of African ancestry compared to Caucasian women. Cancer causes & control: CCC. 2017;28(11):1295–304. 10.1007/s10552-017-0955-2 ; PubMed Central PMCID: PMC5681979. 28887687 PMC5681979

[26] Vermeulen JF, van de Ven RA, Ercan C, van der Groep P, van der Wall E, Bult P, et al Nuclear Kaiso expression is associated with high grade and triple-negative invasive breast cancer. PLoS One. 2012;7(5):e37864 10.1371/journal.pone.0037864 ; PubMed Central PMCID: PMC3360634. 22662240 PMC3360634

[27] Jones J, Wang H, Karanam B, Theodore S, Dean-Colomb W, Welch DR, et al Nuclear localization of Kaiso promotes the poorly differentiated phenotype and EMT in infiltrating ductal carcinomas. Clinical & experimental metastasis. 2014;31(5):497–510. 10.1007/s10585-014-9644-7 ; PubMed Central PMCID: PMC4065802. 24570268 PMC4065802

[28] Elliott B, Zackery DL, Eaton VA, Jones RT, Abebe F, Ragin CC, et al Ethnic Differences in TGFbeta Signaling Pathway May Contribute to Prostate Cancer Health Disparity. Carcinogenesis. 2018 10.1093/carcin/bgy020 . 29474521 PMC5889036

[29] Smith BN, Burton LJ, Henderson V, Randle DD, Morton DJ, Smith BA, et al Snail promotes epithelial mesenchymal transition in breast cancer cells in part via activation of nuclear ERK2. PLoS One. 2014;9(8):e104987 10.1371/journal.pone.0104987 ; PubMed Central PMCID: PMC4133359. 25122124 PMC4133359

[30] Wilder C, Park K, Keegan P, Platt M. Manipulating substrate and pH in zymography protocols selectively distinguishes cathepsins K, L, S and V activity in cells and tissues. Arch Biochem Biophys. 2011;516(1):187–202. 10.1016/j.abb.2011.09.009 PMC3221864 21982919

[31] Burton L, Smith B, Smith B, Loyd Q, Nagappan P, McKeithen D, et al Muscadine grape skin extract can antagonize Snail-cathepsin L-mediated invasion, migration and osteoclastogenesis in prostate and breast cancer cells. Carcinogenesis. 2015;36(9):1019–27. 10.1093/carcin/bgv084 26069256 PMC4643647

[32] Shuch B MM, Satagopan J, Lee P, Yee H, Chang C, Cordon-Cardo C, Taneja SS, Osman I. Racial disparity of epidermal growth factor receptor expression in prostate cancer. J Clin Oncol. 2004;22(23):4725–9. 10.1200/JCO.2004.06.134 15570072

[33] Timofeeva OA ZX, Ressom HW, Varghese RS, Kallakury BV, Wang K, Ji Y, Cheema A, Jung M, Brown ML, Rhim JS, Dritschilo A. Enhanced expression of SOS1 is detected in prostate cancer epithelial cells from African-American men. Int J Oncol. 2009;35(4):751–60. 19724911 PMC3727633

[34] Wallace TA PR, Yi M, Howe TM, Gillespie JW, Yfantis HG, Stephens RM, Caporaso NE, Loffredo CA, Ambs S. Tumor immunobiological differences in prostate cancer between African-American and European-American men. Cancer Res. 2008;68(3):927–36. 10.1158/0008-5472.CAN-07-2608 18245496

[35] Rose AE SJ, Oddoux C, Zhou Q, Xu R, Olshen AB, Yu JZ, Dash A, Jean-Gilles J, Reuter V, Gerald WL, Lee P, Osman I. Copy number and gene expression differences between African American and Caucasian American prostate cancer. J Transl Med. 2010;8(70). 10.1186/1479-5876-8-8 PMC2913940 20649978

[36] Smith BN, Odero-Marah VA. The role of Snail in prostate cancer. Cell adhesion & migration. 2012;6(5):433–41. 23076049 10.4161/cam.21687 PMC3496681

[37] Mikami S, Oya M, Mizuno R, Kosaka T, Ishida M, Kuroda N, et al Recent advances in renal cell carcinoma from a pathological point of view. Pathology international. 2016;66(9):481–90. 10.1111/pin.12433 . 27461942

[38] NickKholgh B, Fang X, Winters SM, Raina A, Pandya KS, Gyabaah K, et al Cell line modeling to study biomarker panel in prostate cancer. The Prostate. 2016;76(3):245–58. 10.1002/pros.23116 ; PubMed Central PMCID: PMC4942245. 26764245 PMC4942245

[39] Ko H, Kim HS, Kim NH, Lee SH, Kim KH, Hong SH, et al Nuclear localization signals of the E-cadherin transcriptional repressor Snail. Cells Tissues Organs. 2007;185(1–3):66–72. 10.1159/000101305 17587810

[40] Rosivatz E, Becker K-F, Kremmer E, Schott C, Blechschmidt K, Höfler H, et al Expression and nuclear localization of Snail, an E-cadherin repressor, in adenocarcinomas of the upper gastrointestinal tract. Virchows Archiv. 2006;448(3):277–87. 10.1007/s00428-005-0118-9 16328348

[41] Goulet B, Sansregret L, Leduy L, Bogyo M, Weber E, Chauhan SS, et al Increased expression and activity of nuclear cathepsin L in cancer cells suggests a novel mechanism of cell transformation. Molecular Cancer Research. 2007;5(9):899–907. 10.1158/1541-7786.MCR-07-0160 17855659

[42] Tamhane T, Illukkumbura R, Lu S, Maelandsmo G, Haugen M, Brix K. Nuclear cathepsin L activity is required for cell cycle progression of colorectal carcinoma cells. Biochimie. 2015. 10.1016/j.biochi.2015.09.003 26343556

[43] De Grotsky. BRCA1 loss activates cathepsin L-mediated degradation of 53BP1 in breast cancer cells. J Cell Bio. 2013;200(2):187–202. 23337117 10.1083/jcb.201204053 PMC3549967

[44] Tedelind S, al e. Nuclear cysteine cathepsin variants in thyroid carcinoma cells. Biol Chem. 2010;391(8):923–35. 10.1515/BC.2010.109 20536394 PMC3518386

