# Supplementary material for: Association of Epithelial Mesenchymal Transition with prostate and breast health disparities
Source: PLoS One. 2018 Sep 10;13(9):e0203855. doi: 10.1371/journal.pone.0203855 (PMC6130866; doi:10.1371/journal.pone.0203855)
Supplement: S1 Table — (DOCX) [file pone.0203855.s001.docx]

| **Prostate** | **African-American**  **(n=74)**  **Normal Cancer**  **(n=36) (n=38)** | **Caucasian**  **(n=78)**  **Normal Cancer**  **(n=33) (n=45)** | **Bahamas**  **(n=56)**  **Normal Cancer**  **(n=34) (n=22)** |
| --- | --- | --- | --- |
| **Snail expression**  **(means)** | **77.886 72.288**    **p=0.3236** | **11.372 70.175**    **p=3.615e-07***** | **24.260 44.349**  **p=0.05622** |
| **Cat L expression**  **(means)** | **71.151 74.148**    **p=0.08747** | **6.966 70.559**    **p=2.674e-07***** | **24.112 45.552**    **p=0.0002003***** |

**S1 Table. Comparison of Nuclear Snail and Cat L in prostate normal vs cancer.**
