# Supplementary material for: Association of Epithelial Mesenchymal Transition with prostate and breast health disparities
Source: PLoS One. 2018 Sep 10;13(9):e0203855. doi: 10.1371/journal.pone.0203855 (PMC6130866; doi:10.1371/journal.pone.0203855)
Supplement: S2 Table — (DOCX) [file pone.0203855.s002.docx]

| Prostate | Condition A | Condition B | Mean A | Mean B | p-value | Significance |
| --- | --- | --- | --- | --- | --- | --- |
| Normal | AA (36) | CA (33) | 77.88618 | 11.37241 | < 2.2e-16 | *** |
| Cancer | AA(38) | CA (45) | 72.28816 | 70.17514 | 0.7637 |  |

**S2 Table. Comparison of nuclear Snail distribution in AA vs CA prostate patients.**
