# Supplementary material for: Association of Epithelial Mesenchymal Transition with prostate and breast health disparities
Source: PLoS One. 2018 Sep 10;13(9):e0203855. doi: 10.1371/journal.pone.0203855 (PMC6130866; doi:10.1371/journal.pone.0203855)
Supplement: S4 Table — (DOCX) [file pone.0203855.s004.docx]

| Breast | Condition A | Condition B | Mean A | Mean B | p-value | Significance |
| --- | --- | --- | --- | --- | --- | --- |
| Normal | AA (24) | CA (10) | 31.16988 | 19.80981 | 0.01601 | * |
| ER+PR+HER2+ | AA(7) | CA (14) | 25.68724 | 22.39063 | 0.3929 |  |
| TNBC | AA (15) | CA (13) | 25.88896 | 15.05352 | 0.01921 | * |

**S4 Table. Comparison of nuclear Snail distribution in AA vs CA breast patients.**
