# Supplementary material for: Association of Epithelial Mesenchymal Transition with prostate and breast health disparities
Source: PLoS One. 2018 Sep 10;13(9):e0203855. doi: 10.1371/journal.pone.0203855 (PMC6130866; doi:10.1371/journal.pone.0203855)
Supplement: S5 Table — (DOCX) [file pone.0203855.s005.docx]

| Breast | Condition A | Condition B | Mean A | Mean B | p-value | Significance |
| --- | --- | --- | --- | --- | --- | --- |
| Normal | AA (24) | CA (10) | 39.72860 | 32.58015 | 0.1141 |  |
| ER+PR+HER2+ | AA(7) | CA (14) | 34.13787 | 38.33871 | 0.5995 |  |
| TNBC | AA (15) | CA (13) | 39.11124 | 37.85855 | 0.9343 |  |

**S5 Table. Comparison of nuclear Cat L distribution in AA vs CA breast patients.**
